# Successful Direct Whole Genome Sequencing and Revivification of Freeze-Dried Nontuberculous Mycobacteria after More than Half a Century of Storage

**DOI:** 10.1128/spectrum.00310-22

**Published:** 2022-05-19

**Authors:** Xenia Emilie Sinding Iversen, Anders Norman, Dorte Bek Folkvardsen, Erik Svensson, Erik Michael Rasmussen, Troels Lillebaek

**Affiliations:** a International Reference Laboratory of Mycobacteriology, Statens Serum Institut, Copenhagen, Denmark; b Global Health Section, Department of Public Health, University of Copenhagen, Denmark; McGill University

**Keywords:** 7 decades, ancient strains, freeze-dried, mycobateria, NGS, revivification

## Abstract

In this study, 28 “historical” clinical freeze-dried nontuberculous mycobacterial isolates collected from 1948 to 1957, were analyzed by investigating their viability and performing whole genome sequencing (WGS) on DNA extracted (i) directly from freeze-dried cells versus (ii) after culturing, to determine cell properties and DNA quality after centuries of freeze-dried storage. The isolated DNA was sequenced on the Illumina MiSeq platform and data quality evaluated analyzing the per-base quality scores of paired-end sequencing reads as well as the overall contiguity of resulting *de novo* assemblies. After 72 years in storage, all freeze-dried isolates were viable, and showed no signs of cell damage and limited signs of contamination when reculturing. They were recultured without problems and identified through WGS with only four of 13 parameters showing statistical significance based on sequence data obtained directly from the freeze-dried cells versus after reculturing, indicating no DNA degradation. Thus, mycobacteria can be whole genome sequenced successfully directly from freeze-dried material without prior recultivation, saving laboratory time and resources, and emphasizing the value of freeze-drying for long-term storage. Our study lays the groundwork for further genomic investigations of freeze-dried bacterial isolates, and the approximately 4,000 historical isolates in our collection will provide a unique opportunity to investigate mycobacterial DNA from a variety of NTM species unexposed to antimicrobials, some maybe still undescribed species.

**IMPORTANCE** The genus Mycobacterium was described more than a century ago and new species are continuously identified and described. There is an ongoing discussion about an increase in the incidence of disease caused by nontuberculous mycobacteria (NTM). How the different bacteria looked before exposure to antibiotics can only be investigated by looking at strains from before the antibiotic era. Strains from that era will be stored in different ways, for example by freeze-drying. The question is how to investigate these strains, and if they are still viable, whether they need to be cultured, and if that changes the DNA. Here, we test all these parameters on freeze-dried strains and show that NGS can be applied directly without culturing.

## INTRODUCTION

The genus Mycobacterium was described over a century ago and by now, it comprises more than 192 species, including several human pathogens (1–3). Among these, Mycobacterium tuberculosis is the most important species and focus of mycobacterial research. For centuries, tuberculosis (TB), the disease predominantly caused by M. tuberculosis, has been a critical threat to public health and even today, it remains the leading cause of death from a single bacterial agent worldwide. Besides mycobacteria causing TB, many nontuberculous mycobacteria (NTM) have been described. NTM are not considered a public health threat like TB, but their importance as opportunistic pathogens is increasingly recognized (4) inspiring new studies of their epidemiology, clinical significance and mechanisms of pathogenicity.

Whole genome sequencing (WGS) of mycobacteria using the Illumina MiSeq platform is widely used in laboratory diagnostics analyzing M. tuberculosis. WGS has become an important tool for identifying antimicrobial resistance predictors and analyzing transmission (5). Reculturing has been considered an essential step to meet the relatively high requirements of DNA quality when performing WGS on mycobacteria (6). However, because mycobacteria are generally slow-growing, and in some cases need to incubate for more than 8 weeks, a reculturing step significantly lengthens this process.

For study purposes, adequate preservation of mycobacterial species is essential. It allows studying the epidemiological and genomic evolution over time, and it has been part of handling clinical bacterial isolates. For decades, a preferred method for long-term storage has been freeze-drying, in which suspensions of highly concentrated isolated cells are frozen, dehydrated through sublimation, and subsequently dried (7). Freeze-drying is done to preserve original cell properties such as appearance and shape (8). The technique requires a cell concentration greater than 10^8^ cells/mL to ensure sufficient surviving cells, as many cells lose viability during the freeze-drying process (9). Freeze-drying is also used for biologically active material such as BCG vaccine to provide a stable bacterial culture without affecting its biological characteristics (10). Properties that make the freeze-drying method efficient are the high rehydration capacity of the microorganisms and the opportunity to reconstitute a successful strain propagation (8).

For several decades, strains of mycobacteria have been collected and stored for characterization and subsequent use in health-related research (7). At the International Reference Laboratory of Mycobacteriology (IRLM), Statens Serum Institut (SSI), Copenhagen, Denmark, clinical mycobacterial isolates have been freeze-dried and stored since 1948. The Danish collection contains over 4,000 mycobacterial strains stored after isolation and identification of routine patient samples. In the early days, the cultivated strains were only identified with Ziehl-Neelsen (ZN) staining and bright field microscopy as being “human” or “saprophyte” before different phenotypic characterization methods became common (11). We investigated the viability, species identity, and general properties of a subset of decade old freeze-dried NTM isolates. Furthermore, we compared the quality of the extracted DNA by using WGS both before and after reculturing the freeze-dried mycobacterial samples.

## RESULTS

### Revival, morphology, viability, and growth characteristics of old freeze-dried mycobacterial isolates.

**FIG 1:**
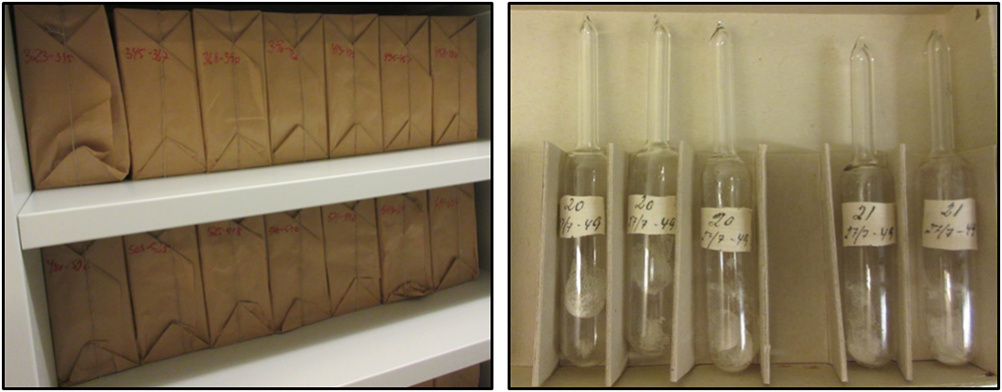
Freeze-dried mycobacterial isolates are stored in glass ampoules packed in boxes and kept at 10°C. The International Reference Laboratory of Mycobacteriology at Statens Serum Institut preserve more than 4,000 freeze-dried isolates received from the 1940s to the 1990s, compromising a historical mycobacteria strain collection.

Since 1949, the mycobacterial strain collection at IRLM at SSI has been stored as freeze-dried isolates inside one or more glass ampoules carefully packed in boxes at constant 10°C (Fig. 1). After opening the glass ampoules, the resuspended mycobacterial isolates all grew well in both liquid (MGIT) and on solid (LJ) medium after 2 to 8 weeks. Thus, all samples were viable after 63 to 72 years in storage. In two of the 28 samples (Mu0057 and Mu0094), some contamination was observed on solid medium, whereas contamination was not apparent in liquid culture.

The historical isolates’ macroscopic morphology was studied on LJ medium, where different colony sizes and pigmentation were observed (Table 1). Some isolates grew fewer and larger colonies, while other isolates grew many small colonies. Both rough and smooth as well as white and transparent colonies were observed. Some colonies also showed yellow pigmentation. The microscopic morphology of resuspended freeze-dried mycobacterial cells was studied using ZN-staining. Here, we observed clumping, rod-shaped mycobacteria with no visible signs of damage to the cells.

**TABLE 1:** NTM species determination performed using multilocus sequence typing of extracted putative coding regions from *de novo* assemblies of all sequenced isolates^*a*^

Isolate		Protein homology to *secA*^*b*^	LJ colony morphology^*c*^
Mu0049		*M. thermoresistibile* (100.0%)	S, t
**Mu0050**		***M. chitae* (93.58%)**	**S, t**
Mu0051		M. avium subsp. *hominissuis* (100.0%)	S, t
**Mu0053**		***M. chitae* (91.84%)**	**S, t**
Mu0054		*M. sinensis* (99.89%)	S, y
Mu0055		*M. algericus* (99.89%)	S, t
Mu0056		*M. algericus* (100.0%)	S, y
Mu0057		*M. algericus* (100.0%)	*-*
Mu0058		*M. algericus* (100.0%)	S, t
Mu0082		*M. algericus* (100.0%)	S, t
**Mu0083**		***M. icosiumassiliensis* (94.91%)**	**R, t**
Mu0084		*M. kumamotonensis* (99.02%)	S, w
Mu0086		*M. branderi* (100.0%)	S, t
Mu0087		*M. nonchromogenicus* (100.0%)	S, t
Mu0088		*M. kumamotonensis* (99.02%)	R, w
Mu0089		*M. sinensis* (99.89%)	S, y
Mu0090		*M. kumamotonensis* (99.02%)	R, w
Mu0091		*M. sinensis* (99.89%)	S, t
Mu0093		*M. novocastrense* (99.68%)	R, y
Mu0094		M. avium subsp. *hominissuis* (100.0%)	-
Mu0100		*M. trivialis* (100.0%)	R, t
Mu0101		*M. nonchromogenicus* (100.0%)	S, t
**Mu0102**		***M. icosiumassiliensis* (98.27%)**	**R, y**
Mu0103		*M. koreensis* (99.68%)	S, t
Mu0132		M. abscessus subsp. *bolettii* (99.57%)	R, w
Mu0134		M. intracellulare subsp. *intracellulare* (100.0%)	S, t
Mu0152		*M. trivialis* (100.0%)	R, t
Mu0971		*M. peregrinum* (98.85%)	R, w

a Protein blast hits of selected marker genes (*secA*, *rpoB*, *rpoC*, *hsp65*, *ileS*, *alaS*, and *leuS*) were used to select 25 genomes as phylogenetic reference points (Table S1 and S2). Colony morphology for isolates were observed from Lowenstein-Jensen cultures. Bold rows define potentially new and undiscovered species. -, no phenotypic characterization due to contamination.

b Amino acid identity of tblastn hits against *secA* against reference genome assemblies downloaded from the NCBI Reference Sequence Database (RefSeq).

c Morphology characteristics: surface: rough (R) or smooth (S); color: transparent (t), white (w), or yellow (y).

### WGS and species identification of mycobacterial isolates.

**FIG 2:**
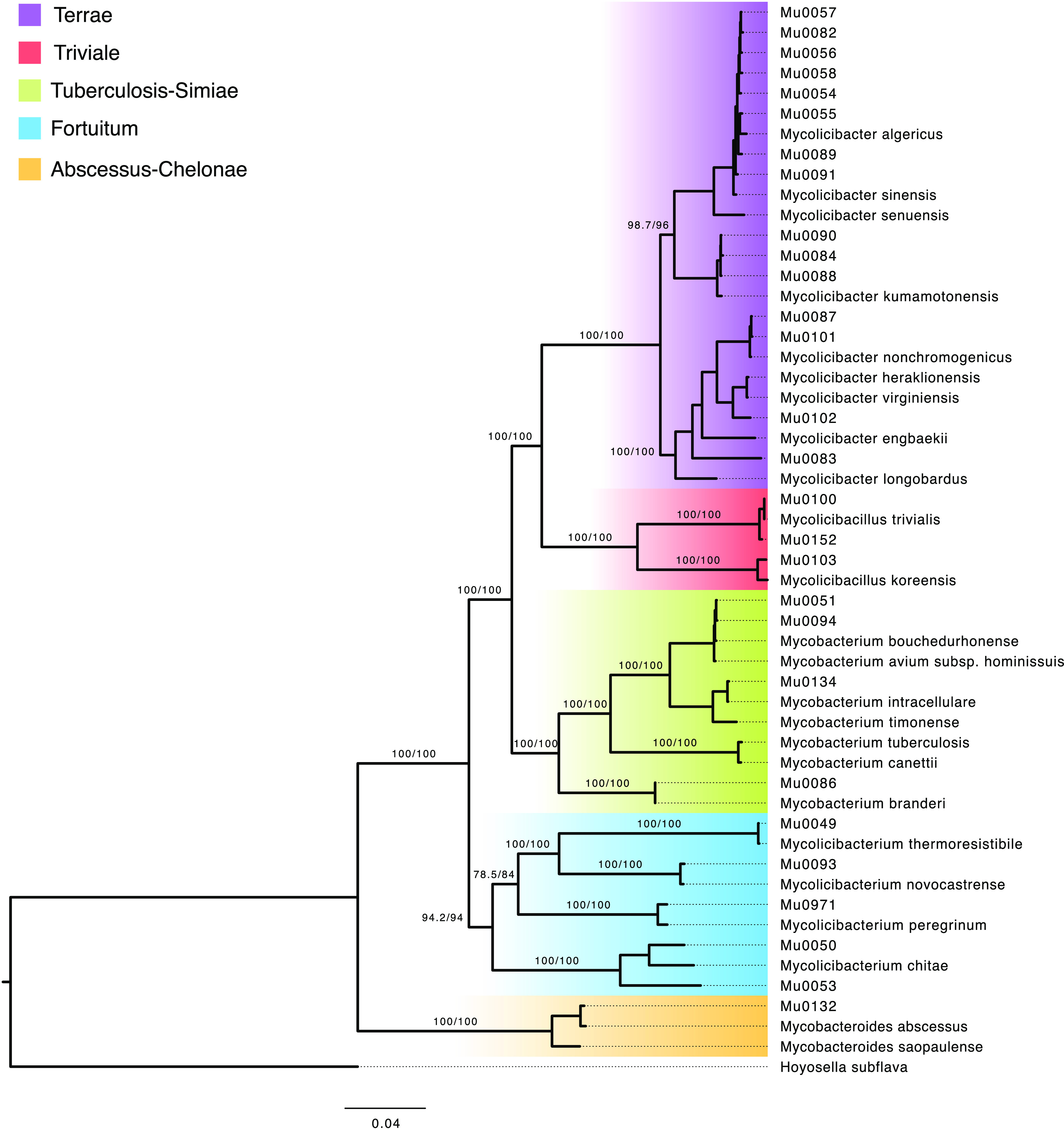
Maximum likelihood phylogeny of 28 assembled historical nontypical mycobacterial isolates and 25 reference genomes downloaded from the NCBI Refseq database, using concatenated protein sequence alignments of 83 single copy core genes. Asterisk signifies isolates that differed by more than 5% from a known reference genome by calculating average nucleotide identities (ANI). Branch support values are ultrafast bootstap (UFBoot) and approximate likelihood ratio test (aLRT) values, respectively.

First, quick k-mer based taxonomic classification of raw sequencing reads was performed to assess the level of contamination with non-mycobacterial species, which could potentially influence downstream analysis (see Supplemental File 1 and File 2 and Table S2). This also resulted in unambiguous species-level identification of 13/28 NTM isolates. For the remaining 15 NTM isolates, core marker genes extracted from *de novo* genome assemblies were used to assign 19/20 of the freeze-dried NTM isolates to known mycobacterial species (see Supplemental File 2, Table S3, Table S4, and Table S5). Then, a robust phylogeny using a concatenated protein alignment consisting of 83 different core gene products was constructed (Fig. 2).

Upon inspection of the phylogenetic tree, it was observed that the 28 historical isolates were represented across five clearly distinct clades within the family Mycobacteriaceae. Gupta et al. (12) previously designated these Terrae, Triviale, Tuberculosis-Simiae, Fortuitum, and Abscessus-Chelonae, respectively, which is also indicated in Fig. 1. Phylogenetic placement based on core genes was entirely consistent with the assigned species based on marker genes for 24 of the 28 isolates, and unassigned isolates appeared appropriately distant from their respective references. However, one more isolate (Mu0102) seemed to differ significantly from known NTM species but was most closely associated with *M. heraklionensis* and *M. virginiensis*. Two of our putative unassigned NTM isolates (Mu0050 and Mu0053) were related to species within the Fortuitum clade, while the other two (Mu0083 and Mu0102) belong to the Terrae clade.

### Comparing WGS of uncultured and cultured isolates using principal component analysis.

**FIG 3:**
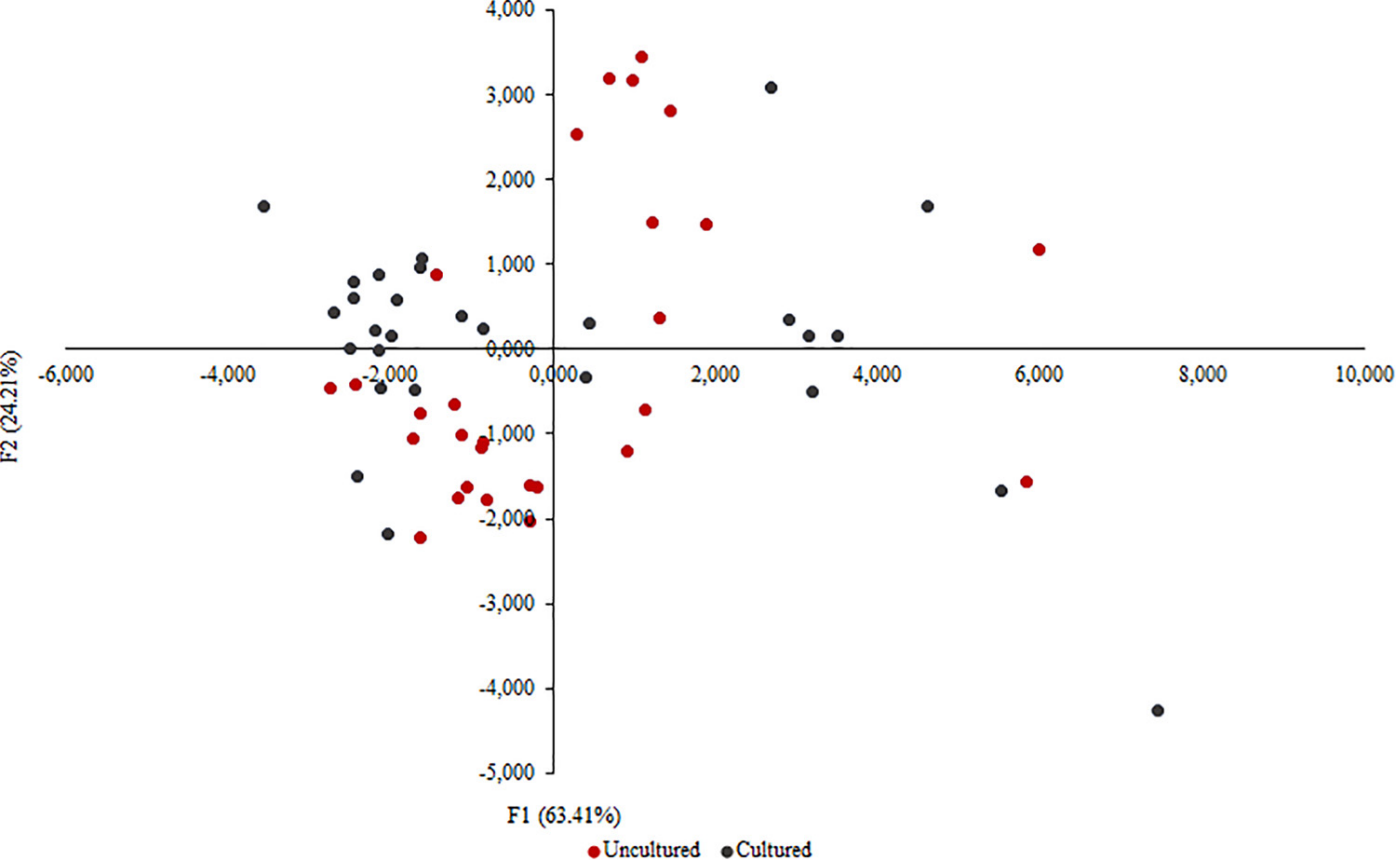
Principal component analysis (PCA) and observation chart based on 13 mapping parameters obtained from samtools was made using XLSTAT (v22.2.2) to compare sequencing of uncultured and cultured isolates. The chart is created based on the eigenvalues of factor F1 (63.41%) and F2 (24.21%) of the initial data set, presenting 87.62% of the initial data set.

The extraction of uncultured and cultured freeze-dried NTM samples was compared using the 13 extracted parameters from the samtools analysis and resulted in almost equal outcome with only minimal deviations (Table 2). The median of the number of sequence reads, total of bases sequenced, and insert sizes of mapped reads were similar for both uncultured and culture samples (*P*-values = 0.60, 0.62, and 0.51, respectively; two-tailed T-test) (Table 2). A significant difference between the methods was calculated using a paired two-tailed T-test to compare the two methods at a statistical level. Only the four parameters percentages of mismatches per mapped base, Q-score on forward reads, Q-score on reverse reads, and upper 95% limit of insert sizes, showed a statistically significant difference between the two methods (*P* = ≤ 0.01; two-tailed T-test). The 10 nonpercentage parameters were used for principal component analysis (PCA) and to make an observation chart for summation and visualization. The PCA observation chart presented 87.62% of the initial variability of the original data set, ensuring good analytical quality. Furthermore, a merged distribution of uncultured and cultured samples was observed, thereby indicating unambiguous sequencing method results, supporting the observed tendency when calculating median and statistical differences (Fig. 3).

**TABLE 2:** Statistical comparison of the Illumina MiSeq run data-output parameters for uncultured and cultured samples, obtained from samtools analysis^*a*^

Illumina run parameters	Median Uncultured samples	Median Cultured samples	T-test *P*-value
No. of sequence reads (forward and reverse combined)	1,332,293	1,638,481	0.60
Total no. of bases sequenced	161,258,608	201,940,270	0.62
Avg. coverage of mapped bases over contigs	27	31	0.90
Percentage of sequenced bases mapped to contigs	71%	70%	0.71
Percentage of mismatches per mapped base (error rate)	**0.22%**	**0.32%**	**< 0.01** ^*b*^
Percentage of sequenced bases with a quality score ≥ Q30 on forward reads	**97%**	**95%**	**< 0.01** ^*b*^
Percentage of sequenced bases with a quality score ≥ Q30 on reverse reads	**96%**	**93%**	**< 0.01** ^*b*^
Mode of the distribution of insert sizes mapped reads	89	110	0.07
Median of insert sizes of mapped reads	259	233	0.51
Lower Quartile (Q1) value of insert sizes	166	155	0.80
Upper Quartile (Q3) of insert sizes	392	335	0.07
Lower 5% limit of insert sizes	81	83	0.40
Upper 95% limit of insert sizes	**609**	**519**	**< 0.01** ^*b*^

a The median as well as a paired two-tailed T-test was calculated for the data-output parameters using Excel (v15.32), where parameter data from uncultured sample runs and cultured sample runs were chosen as vector 1 and vector 2, respectively. Significant difference between the two methods (*P* = ≤0.01) is defined in bold.

b Significant *P*-value < 0.05.

## DISCUSSION

In the present study, we selected 28 historical 63- to 72-year-old mycobacterial isolates from the IRLM strain collection to investigate the viability and perform WGS on these long-term stored freeze-dried bacterial isolates. We document that it is possible to revive and fully sequence all 28 genomes directly from freeze-dried samples and from subsequent cultures. Following the revivification, ZN microscopy proved no visible sign of cell lysis.

Previous studies have reported freeze-dried bacteria will remain viable for at least 33 to 39 years after freeze-drying, provided a sufficient amount of bacteria was added to the vials. In the present study, we demonstrate viability after almost twice as long, which, to our knowledge, is the longest documented time period of mycobacterial freeze-drying with a subsequent successful revival (13–15).

WGS could be performed successfully for both cultured and uncultured freeze-dried isolates. Results showed that sequencing the uncultured freeze-dried samples was equally efficient to sequencing the cultured samples. To see whether the sequence results were different, in one run, both cultured and uncultured samples were sequenced on the same flow cell on the Illumina MiSeq platform, but no differences were observed. Due to the natural mutation rate, there might occur slight differences in the genome sequences between the cultured and the uncultured samples. However, it seems unlikely, that these single nucleotide polymorphisms (SNPs) would have a major impact on the interpretation of the genome sequence analysis as has been suggested by Kucukyildirim et al. and Schnappinger and Ehrt (16, 17). Even though four of 13 data output parameters showed a significant difference (*P < *0.01; two-tailed T-test) between the two presequencing methods (Table 2), these did not affect the unambiguous results when analyzing and comparing genomes of cultured and uncultured isolates and where therefore considered not crucial. To our knowledge, this study is the first of its kind to describe WGS directly from re-suspended freeze-dried mycobacteria isolates.

Our study identified common species such as M. avium and M. intracellulare, typically related to pulmonary NTM diseases today, but also several less common species such as *M. thermoresistibile* and *M. nonchromogenicus* (18, 19). Through protein homology and phylogenetic analyses, we observed that the sequenced isolates were distributed over five distinct groups of the taxonomic family Mycobacteriaceae, equivalent to the groups described by Gupta et al. (12), and found four revived isolates that differed significantly from any hitherto known sequenced NTM species. These four NTM isolates, which group within the Terrae and Fortuitum clades, are potentially new and undiscovered species, although deeper analyses are necessary to draw any further conclusions. As these four unknown NTM species have not been described or sequenced before, they may be particularly rare in a contemporary clinical context. Maybe these strains were also unknown when they were isolated, and not possible to characterize with techniques available at the time of collection and were perhaps stored for future analysis and identification.

Several methods for the long-term preservation of bacterial isolates have been developed through time, and one approach is to preserve the isolates frozen in glycerol stocks. Previous studies show that the glycerol content may negatively affect the bacteria, leading to a nonefficient way to store bacterial isolates for long-term preservation (6). Freeze-drying is an effective but intricate method, as the many physical and biological factors inflicted on the bacteria by this method may significantly impact its survival. It is essential to maintain viability during the freeze-drying laboratory procedure and the storage, which should be considered during the process (8). Nevertheless, in this study, we suggest that the freeze-drying method is preferable for the long-term preservation of mycobacterial isolates. The isolates can be sequenced directly from a freeze-dried state with no need for cultivation. Furthermore, storage of freeze-dried strains is more efficient as the isolates are kept at 10°C and not easily influenced by power cuts, compared with e.g., glycerol storage that requires −80°C for optimal storage. This point should be taken into account when storing isolates for decades. The IRLM collection of more than 4,000 mycobacterial isolates, freeze-dried and stored since the 1940s, could give a picture of important evolutional and pathological perspectives to understand the history of mycobacteria species.

A potential limitation to this study is that the freeze-dried bacteria were cultured before freeze-drying, which might pose a disadvantage as modern molecular studies have documented the rare occurrence of polyclonal infections with multiple mycobacterial strains within a host (20). Thus, we might potentially overlook other less frequent variants.

In conclusion, in this study we show that mycobacteria can be successfully whole genome sequenced directly from the freeze-dried material without the need for prior recultivation. When comparing Illumina MiSeq runs, we observed no significant differences with respect to the quality in sequence data between the two methods that could not also be explained by the batch effect. Studying our historical strain collection containing approximately 4,000 mycobacteria isolates will provide an unique opportunity to investigate “historical” mycobacterial DNA from before many NTM species were first described and also, before they were exposed to antimicrobials. Our study indicates that freeze-drying of mycobacterial isolates is an appropriate method for long-term storage, as it retains the quality and properties of the bacteria that are still viable after 72 years of storage. In addition, freeze-dried isolates can be whole genome sequenced directly without recultivation, lowering the possibility of laboratory cross contamination and preserving the original genetic information.

## MATERIALS AND METHODS

### Selection of historical isolates.

The vast majority of isolates are based on specimens from humans, and for this study, 28 freeze-dried mycobacterial isolates collected from different patients born between the years 1893 and 1940 were selected, with an equal distribution between males and females. These isolates, listed as saprophytes in the original strain collection metadata, were pulmonary, originating from either sputum or gastric lavage samples collected from 1948 through 1957. The selection was based on the age of the samples (the older ones preferred), the availability of associated historical meta-data, and availability of at least two ampoules stored per sample in order not to use the last available ampoule for this proof-of-concept study. All experimental procedures on live bacteria were performed within the BSL-3 classified laboratory harboring the historical strain collection.

### Preparation, culturing, and microscopy.

The ampoules with the freeze-dried pellets were opened carefully, and the isolates were suspended in Dubos medium (see Supplemental File 1). Each cell suspension was divided into two fractions, one for direct DNA isolation and one for re-culturing. All 28 isolates for re-culturing were inoculated in both Bactec Mycobacteria Growth Indicator Tubes (MGIT; Bactec MGIT 960 Mycobacterial Detection System, BD Diagnostic Systems, Franklin Lakes, NJ, USA) and on solid Löwenstein-Jensen slants (LJ; SSI Diagnostica, Hilleroed, Denmark) for up to 8 weeks before DNA isolation. ZN staining on two drops of well-mixed isolate suspension was also performed to examine the freeze-dried cells using light microscopy (see Supplemental File 1).

### DNA preparation and illumina sequencing.

The cultured mycobacterial isolates and suspended freeze-dried isolates all followed the same DNA isolation procedure using the QIAmp DNA minikit (Qiagen, Hilden, Germany) protocol with minor modifications to optimize for mycobacterial DNA extraction (see Supplemental File 1). DNA concentrations were determined using Qubit dsDNA HS assay kit (0.2 to 100 ng) and the Qubit 2.0 Fluorometer according to the manufacturer’s instructions (Life Technologies, Thermo Fisher Scientific Inc., Waltham, MA USA), to obtain a final DNA concentration at 0.2 to 0.4 ng/μL. Libraries were prepared using Nextera XT DNA Library Preparation Kit (Íllumina, San Diego, CA, USA) according to the manufacturer’s protocol. Libraries were sequenced using the MiSeq sequencing platform (Illumina). The MiSeq sequencing was carried out using the MiSeq reagent kit v2 300 Cycles (Illumina; 2 × 150 bp read length).

### Taxonomic read classification.

The program Bracken (v2.2) was used to estimate the species-level composition of each sample by evaluating taxonomically classified reads from the output of the program Kraken (v2.07; K-mer database used: minikraken2_v2_8GB_201904). The bracken output was simplified by combining identified species into the following three taxonomic groups; M. tuberculosis *complex* (MTBC; M. tuberculosis and *M. canettii* only), NTM (all mycobacteria not belonging to MTBC), and non-mycobacteria, which included all other species not belonging to the two former groups.

### *De novo* genome assembly and read mapping.

To prepare reads for *de novo* assembly, residual adapter fragments were removed, and quality-trimmed paired-end Illumina reads using the program Trimmomatic (v0.39) with the trimming option string ILLUMINACLIP:NexteraPE-PE.fa:2:8:24 SLIDINGWINDOW:4:13 MINLEN:36 AVGQUAL:25. SPAdes software (v3.13.1) was used for each sample to assemble reads with the–isolate option enabled, which is a workflow optimized for high coverage bacterial isolate data. Each of the respective sequenced cultured and uncultured sample libraries was first assembled by itself and then pooled into one combined hybrid assembly so that each sample was represented by three different *de novo* assemblies (i.e., uncultured, cultured, and pooled). Then, contigs with less than half of the average read depth than the most common read depth of the assembly were removed, to ensure the exclusion of contigs assembled from low-abundance contaminating reads. The tool QUAST (21) was then used to evaluate the quality of each assembly and select the assembly that produced the highest N50, defined as the shortest contig length needed to cover 50% of the length of the total assembly. In all but two instances, pooling of libraries produced the best assembly. Reads from both libraries were mapped back to the contigs of the winning assembly from their respective sample, using the Burrows-Wheeler aligner bwa (v0.7.17), and de-duplicated using picard-tools (v2.12). Samtools (v1.9) was used to extract all relevant mapping parameters, such as base qualities, error rates, and insert sizes from BAM-formatted read mapping files (used below).

### Statistical methods.

In total, 13 parameters were used to compare runs of uncultured and cultured isolates. Excel (v15.32) was used to calculate the median for all parameters of uncultured and cultured isolates, respectively, and to calculate a statistical difference between the two prior-sequencing methods using a paired two-tailed T-test or Fisher’s exact test. Furthermore, a PCA was done using XLSTAT (v22.2.2), a statistical software package for Microsoft Excel, to visualize and analyze correlations between the variable methods.

### Species identification through core gene phylogenetic analysis.

Phylogenetic core marker genes from each genome assembly were extracted using a modified version of the method outlined in Na et al. (22) (see Supplemental File 1). Then, this core set was used to perform translated nucleotide blast (tblastn) against mycobacterial reference genome assemblies available in the NCBI Refseq database, using only the six largest translated marker genes of mycobacterial genomes (*alaS*, *ileS*, *leuS*, *rpoB*, *rpoC*, and *secA*) and the widely used mycobacterial marker gene *hsp65* (13). This analysis was used to identify a set of 25 closely related reference genomes, which were included in our phylogenetic analysis. Protein sequences were aligned separately with MAFFT, using the option–auto. The program Gblocks (v0.91b) was used to remove poorly aligned and phylogenetically unstable regions and then concatenated the remaining alignments before phylogenetic analysis. Phylogenetic trees were constructed using the program IQ-tree (v1.6.12) using the evolutionary model LG+F+R11, which scored highest according to the Bayesian information criterion using IQ-tree’s built-in model selection function. Branch support values were calculated using 1,000 ultrafast bootstraps (-bb) and 1,000 SH-like aLRT bootstraps (-alrt).

### Approval.

This method study was based on a mycobacterial strain collection and did not include any patient information. Thus, approvals were not required according to Danish law.

### Data availability.

Illumina sequences have been deposited in the European Nucleotide Archive under project accession number PRJEB50291.
