# Supplementary material for: Successful Direct Whole Genome Sequencing and Revivification of Freeze-Dried Nontuberculous Mycobacteria after More than Half a Century of Storage
Source: Microbiol Spectr. 2022 May 19;10(3):e00310-22. doi: 10.1128/spectrum.00310-22 (PMC9241853; doi:10.1128/spectrum.00310-22)
Supplement: SUPPLEMENTAL FILE 1 — Supplemental material. Download spectrum.00310-22-s001.pdf, PDF file, 0.9 MB [file spectrum.00310-22-s001.pdf]

Supplement

Successful direct whole genome sequencing and revivification of freeze-dried nontuberculous mycobacteria after more than half a century of storage

Xenia Emilie Sinding Iversen, Anders Norman, Dorte Bek Folkvardsen, Erik Svensson, E. Michael Rasmussen, Troels Lillebaek

Material and Methods

Opening of freeze-dry ampoules

To access the freeze-dried cell pellets, ampoules were opened individually, by first using a small saw blade to score around the top. Secondly, a piece of paper moistened with 70% ethanol was applied to cover the upper half, allowing for safe snapping of the ampoule without releasing freeze-dried bacterial powder inside the biosafety cabinet. The isolates were suspended in Dubos medium and each cell suspension was divided into two fractions, one for direct DNA isolation and one for re-culturing.

ZN staining procedure

Two droplets of well-mixed isolate solution were added onto a glass slide and placed to air-dry the smear and then heat fixed using a Bunsen burner. The glass slide was placed on a rack, and the sample solution was covered with a blotting paper before flooded with 5 to 6 droplets of the primary stain carbol fuchsin. The sample was again heated using a Bunsen burner and left at room temperature for 5 minutes, and afterward, the blotting paper was removed and the underside of the slide was rinsed with water to wash off the stain. The glass slide was then placed in an 0.7 % acid-alcohol solution consisting of hydrochloric acid and ethanol for 4 minutes, rinsed with water and placed in a methylene blue solution composed of 2.4 g methylene blue

(USP), 300 ml ethanol, and 700 ml distilled water for 30 seconds. The slide was rinsed with water and placed to air-dry before microscopically examination using a light microscope with 100x oil immersion objective.

DNA extraction

In preparation for WGS, the majority of the re-cultured isolates (n = 25) were retrieved from liquid growth medium, while the remaining (n = 3) were collected from solid medium to ensure sufficient DNA concentration for sequencing. Additionally, DNA was recovered directly from the Dubos suspension of freeze-dried material. We followed the same laboratory procedures during DNA extraction, library preparation and WGS for both cultured- and uncultured isolates. The cultured mycobacterial isolates, as well as suspended freeze-dried isolates, all followed the same DNA isolation procedure using the QIAmp DNA mini kit (Qiagen, Hilden, Germany) protocol with following modifications to optimize for mycobacterial DNA extraction: Samples were centrifuged for 10 minutes; the supernatant was discarded from the tubes and the bacterial pellet was resuspended in 200 μl RNase-free water. The sample solutions were vortexed for 30 seconds, spun down and cells were heat-inactivated at 80°C for 30 minutes. Hereafter, the samples were cooled at room temperature for 5 minutes, spun down, and vortexed for 5 minutes, followed by the addition of 130 μl lysozyme mix consisting of 20 mg/ml lysozyme, 88 % TRIS-buffer (20 mM Tris/HCL pH 8.0/ 2mM EDTA) and 12% Triton X-100 (1.2 % Triton X-100) and 20 μl proteinase K were added before incubating at 56°C for 90 minutes, after which, each sample was shaken 15 minutes. Samples were spun down, and added 200 μl AL lysis buffer, vortexed, spun down and incubated at 70°C for 10 minutes. Hereafter, the samples were cooled at room temperature for 5 minutes, spun down and added 200 μl 99.5% ethanol before transferred to spin columns (QIAamp Spin Columns) and centrifuged for 1 minute at 6,000 rcf. Spin columns were placed in new collecting tubes, washed with 500 μl AW1 and centrifuged for 1 minute at 6,000 rcf. Again, spin columns were placed in new collecting tubes, washed with 500 μl AW2 and centrifuged for 3 minutes at 20,000 rcf. Collecting tubes were replaced with new ones and the samples centrifuged for 1 minute at 20,000 rcf. The

spin columns were transferred to new Eppendorf tubes and added 50 μl 10 mM Tris-cl dilution followed by incubation at room temperature for 10 minutes and centrifugation for 1 minute at 6,000 rcf.

The DNA yields from cell suspensions of uncultured freeze-dried mycobacteria and cell cultures did not differ significantly (Table S1; range 0.112 ng/µL – 0.505 ng/µL, $p = 0.32$; two-tailed T-test).

Sample contamination and species-level assignment from taxonomically classified Illumina sequencing reads

Firstly, k-mer based species classification of sequencing reads from the kraken/bracken analysis showed that all 28 samples consisted almost entirely of NTM reads (median 99.4%, IQR 96-100%) (Table S2). The overall composition showed no significant differences between cultured and directly sequenced samples ($p = 0.12$; Fisher's exact test). The proportion of MTBC classified reads did not exceed 4% (median 0.0%, IQR 0.0-0.9%), while non-mycobacterial reads did not exceed 11.2% (median 0.5%, IQR 0.12-2.6%). Culturing freeze-dried samples seemed to slightly lower the proportion of non-mycobacteria reads (median non-mycobacteria 0.2%, IQR 0.1-1.6% vs. 0.7%, IQR 0.3-4.0%), which could, theoretically, stem from a presence of non-viable bacteria in the freeze-dried pellet. However, this was not statistically significant ($p = 0.63$; Fisher's exact test) and was not investigated further. The following NTM-species could unambiguously be assigned to samples, based on a composition of 90% or higher of reads assigned to a single representative genome: *M. sinensis* (n=8), *M. avium* (n=2), *M. intracellulare* (n=1), *M. abscessus* (n=1), and *M. thermoresistibile* (n=1). The difference of species-level classified reads between cultured and freeze-dried preparations was less than 1% in these 13 samples. The remaining 15 isolates had reads assigned to multiple different NTM species, and showed a higher degree of non-Mycobacterial reads, and could therefore not be readily identified. We understand this as a lack of a suitable representative genomes in the k-mer training data, leading to spurious species classifications which might otherwise wrongly be interpreted as highly heterogeneous species compositions.

Core marker gene extraction from genome assemblies

We used a modified version of the procedure outlined in Na *et al*[1] to identify a suitable set for a core gene phylogeny. Briefly, this involves identifying all putative coding regions (CDSs) in a genome assembly, using the program prodigal, and then scanning the encoded protein sequences for conserved protein domains using hmmscan, which is part of the HMMER software package (v3.12b). Na *et al* defined a set of 92 universally conserved single-copy core genes, suitable for core-gene phylogenies, termed UBCG and provide the HMMER database UBCG.hmm (https://www.ezbiocloud.net/tools/ubcg), which we used in our analysis. Additionally, we included the HMM-profile TIGR02348, extracted from the TIGRFAMs database release 15.0 using hmmfetch, to capture the *hsp65* marker gene encoding a 65 kDa GroEL-like chaperonin protein, widely used to identify NTM[2]. In an attempt to resolve the remaining ambiguous species delineations, we selected a subset of 25 genomes from a set of 192 available reference assemblies belonging to the taxonomic family Mycobacteriaceae (Table S4) to serve as phylogenetic reference points. In total, we included 82 out of the 92 UBCG core genes (Table S5), which were represented over all 28 assemblies and 25 NTM reference genomes. As before, we also included the common mycobacterial phylogenetic marker gene *hsp65*.

Table S1

Input DNA concentrations prior Illumina MiSeq runs and run number of uncultured and cultured samples are present for each isolate, showing sufficient amount of input DNA as well as plate content of either uncultured- (run 1 and 4), cultured- (run 3, 5, and 6), or mixed samples (run 2).

	**Uncultured**		**Cultured**	
**Isolate**	**DNA concentration (ng/µl)**	**Run No.**	**DNA concentration (ng/µl)**	**Run No.**
**Mu0049**	0.38	1	0.45	2
**Mu0050**	0.19	1	0.24	2
**Mu0051**	0.22	1	0.35	2

**Mu0053**	0.27	1	0.21	5
**Mu0054**	0.11	1	0.40	2
**Mu0055**	0.31	1	0.14	2
**Mu0056**	0.14	1	0.11	2
**Mu0057**	0.20	4	0.51	5
**Mu0058**	0.16	4	0.18	5
**Mu0082**	0.23	4	0.17	5
**Mu0083**	0.35	4	0.35	5
**Mu0084**	0.24	4	0.17	5
**Mu0086**	0.33	4	0.46	5
**Mu0087**	0.40	4	0.14	5
**Mu0088**	0.28	4	0.48	5
**Mu0089**	0.30	4	0.30	5
**Mu0090**	0.13	4	0.21	5
**Mu0091**	0.20	4	0.28	5
**Mu0093**	0.32	4	0.36	6
**Mu0094**	0.35	1	0.11	5
**Mu0100**	0.12	4	0.37	5
**Mu0101**	0.40	4	0.19	5
**Mu0102**	0.12	4	0.37	5
**Mu0103**	0.35	4	0.38	5
**Mu0132**	0.42	2	0.12	2
**Mu0134**	0.12	2	0.32	3
**Mu0152**	0.26	4	0.20	5
**Mu0971**	0.20	2	0.42	3

References

1. Na, S. I. *et al.* UBCG: Up-to-date bacterial core gene set and pipeline for phylogenomic tree reconstruction. *J. Microbiol.* **56**, 281–285 (2018).

2. Escobar-Escamilla, N. *et al.* Hsp65 Phylogenetic Assay for Molecular Diagnosis of Nontuberculous Mycobacteria Isolated in Mexico. *Arch. Med. Res.* **45**, 90–97 (2014).
